# Altered Functional Network in Infants With Profound Bilateral Congenital Sensorineural Hearing Loss: A Graph Theory Analysis

**DOI:** 10.3389/fnins.2021.810833

**Published:** 2022-01-14

**Authors:** Wenzhuo Cui, Shanshan Wang, Boyu Chen, Guoguang Fan

**Affiliations:** Department of Radiology, The First Affiliated Hospital of China Medical University, Shenyang, China

**Keywords:** functional connectivity, graph theory, hub, infants, resting-state functional magnetic resonance imaging, sensorineural hearing loss

## Abstract

Functional magnetic resonance imaging (fMRI) studies have suggested that there is a functional reorganization of brain areas in patients with sensorineural hearing loss (SNHL). Recently, graph theory analysis has brought a new understanding of the functional connectome and topological features in central neural system diseases. However, little is known about the functional network topology changes in SNHL patients, especially in infants. In this study, 34 infants with profound bilateral congenital SNHL and 28 infants with normal hearing aged 11–36 months were recruited. No difference was found in small-world parameters and network efficiency parameters. Differences in global and nodal topologic organization, hub distribution, and whole-brain functional connectivity were explored using graph theory analysis. Both normal-hearing infants and SNHL infants exhibited small-world topology. Furthermore, the SNHL group showed a decreased nodal degree in the bilateral thalamus. Six hubs in the SNHL group and seven hubs in the normal-hearing group were identified. The left middle temporal gyrus was a hub only in the SNHL group, while the right parahippocampal gyrus and bilateral temporal pole were hubs only in the normal-hearing group. Functional connectivity between auditory regions and motor regions, between auditory regions and default-mode-network (DMN) regions, and within DMN regions was found to be decreased in the SNHL group. These results indicate a functional reorganization of brain functional networks as a result of hearing loss. This study provides evidence that functional reorganization occurs in the early stage of life in infants with profound bilateral congenital SNHL from the perspective of complex networks.

## Introduction

Sensorineural hearing loss (SNHL) is caused by lesions in the hair cells, vestibulocochlear nerve, or auditory cortex of the brain. In children, the causes of SNHL are not completely understood, but genetic factors may play a part (Kvestad et al., 2014). Cochlear implantation (CI) is an important procedure for the restoration of hearing for children with severe-to-profound SNHL. The United States Food and Drug Administration (FDA)’s recommended age of CI is at least 12 months (Purcell et al., 2021). One study found that children with congenital SNHL benefit more if they receive CI at the age of 1–3 years (Zwolan et al., 2004). Another study suggested that children with congenital SNHL who receive CI before the age of 3.5 benefit more than those who receive it after the age of seven (Sharma and Campbell, 2011). In humans, there is a rapid microstructural white matter development during the first 3 years of life (Lebel and Deoni, 2018). Therefore, the age before 3.5 years is considered as the sensitive or critical period, and the age of 7 as the end of this period (Sharma et al., 2015; Patton et al., 2019). During this period, the auditory cortex has maximum plasticity, and children with congenital hearing loss can benefit the most from CI.

Patients with SNHL need to undergo magnetic resonance imaging (MRI) examinations before CI to confirm whether there are lesions in the central neural system or malformations in the inner ear. In recent years, functional magnetic resonance imaging (fMRI) has been used to estimate the functional changes in the brain in SNHL patients. Task-based fMRI studies found that the auditory areas of SNHL patients can be activated by visual and somatosensory stimuli due to long-term hearing deprivation, which is usually called cross-modal reorganization (Finney et al., 2001; Cardon and Sharma, 2018). Seed-based functional connectivity studies also found alterations in auditory, visual, and language areas (Heggdal et al., 2016; Shi et al., 2016). Furthermore, independent component analysis (ICA) based on resting-state functional magnetic resonance imaging (rs-fMRI) research showed dysconnectivity within and between multiple resting-state networks of executive control, higher-order cognition, and attention (Luan et al., 2019), indicating that the effects of SNHL are not limited to only several systems and resting-state networks but may result in changes in the whole brain network. The study of Stolzberg et al. on neonatal deaf cats using ICA method also found widely altered functional connectivity in both auditory and non-auditory regions (Stolzberg et al., 2018). However, evidence also showed different cross-modal reorganization pattern in primary and high-order auditory cortex (Kral et al., 2003; Berger et al., 2017).Therefore, the changes in the whole-brain functional network in SNHL patients are worth studying.

Complex network analysis, which originates from graph theory, has been used to characterize the functional connectomes within the whole-brain network (Benito-León et al., 2019). This approach provides a topological method to describe network properties (Rubinov and Sporns, 2010) and could be a good complement to seed-based functional connectivity. In recent years, the brain structural and functional network has been found to be changed in many neurological and psychological disorders (Suo et al., 2017a,b, 2019; Benito-León et al., 2019; Openneer et al., 2020; Radetz et al., 2020). Therefore, complex network analysis may be a new approach to evaluating brain functional plasticity in SNHL. Using graph theory analysis, Bonna et al. (2021) found reduced modularity and disrupted functional connectivity in adults who became deaf in childhood when compared with normal hearing siblings. Li et al. (2016) found altered functional connectivity and hub distribution in SNHL adolescents. However, the changes in topological properties and whole-brain functional connectivity in congenital SNHL infants within the developing critical period remain unknown.

In the present study, we used graph theory analysis to compare the alterations in the functional connectome in bilateral congenital SNHL infants and normal-hearing infants within the critical period. Based on previous evidence from both neuroimaging and clinical studies, we hypothesized that the functional network topology of SNHL infants would have similar alterations to that in adults, compared to normal-hearing siblings. We also hoped that this study could provide a new understanding of neuroplasticity in bilateral congenital SNHL infants within the critical period. The University of North Carolina (UNC) 2-year-old infant atlas (Shi et al., 2011) based on Automated Anatomical Labeling (AAL) was used to improve the accuracy of the analysis of infant brain images.

## Materials and Methods

### Participants

This study included 38 infants with congenital bilateral SNHL and 31 with normal hearing. All SNHL infants failed to pass the newborn hearing screening examinations after birth. The auditory brainstem response (ABR) results were symmetrically greater than 90 dB, indicating profound hearing loss. These infants were candidates for cochlear implant surgery, and an MRI scan was used as a presurgical evaluation. None of the patients indicated a history of head surgery, ototoxic drug use, trauma, or any central nervous system disease. The participants of SNHL group had no history of wearing hearing aids. In addition, high-resolution computed tomography (HRCT) scans showed no inner ear malformation. In the normal-hearing (NH) group, participants underwent MRI examinations for non-hearing indications, and MRI scans showed no central nervous system lesions. Infants in the NH group had no history of neurological disease, head injury, surgery, or hearing problems. The data of two SNHL infants and one NH infant were excluded from the analysis due to excessive head movement during scanning. Another two SNHL infants were excluded because they did not finish the scan. Consequently, 34 infants with congenital bilateral SNHL (16 males and 18 females; mean age 23.41 ± 8.26 months; age range 11–36 months) and 30 infants with normal hearing (16 males and 14 females, mean age 24.03 ± 7.18 months; age range 11–36 months) were included in this study. The SNHL group and NH group were matched in age (*p* = 0.089) and gender (chi-squared *p* = 0.616). All the examinations were approved by the ethics committee of the hospital. Informed consent was obtained from every subject’s parent.

### Data Acquisition

MR images were collected on a Siemens Verio Tim 3.0 T MR scanner (Siemens Medical Solutions, Erlangen, Germany) with a 16-channel head coil. To acquire resting-state fMRI data, all the subjects were sedated with 50–60 mg/kg of 10% chloral hydrate orally 15 min before scanning. Earplugs and headphones were used to protect hearing and reduce the impact of scanner noise. Infants were observed closely by a pediatrician during the scans. Anatomical MRI and fMRI acquisitions were obtained from all infants using the protocol detailed below.

Routine MRI scans consisted of axial and sagittal T1-weighted images (T1WI) and axial and coronal T2-weighted images (T2WI) to observe whether there were lesions and/or abnormalities in the brain. After that, the echo-planar imaging sequence was used to acquire resting-state fMRI data. The parameters were as follows: repetition time (TR), 2000 ms; echo time (TE), 30 ms; field of view (FOV), 220 mm × 220 mm; flip angle, 90°; slice thickness, 4 mm; matrix, 70 × 70; voxel size, 3.1 mm × 3.1 mm × 4 mm; 30 slices were acquired covering the whole brain, and the total volume was 190. High-resolution T1-weighted structural brain images were collected using a 3D SPGR sequence with the following parameters: TR, 2400 ms; TE, 3.16 ms; inversion time (TI), 900 ms; FOV, 220 mm × 220 mm; flip angle, 9°; slice thickness, 1 mm; matrix, 224 × 256; voxel size, 1.0 mm × 1.0 mm × 1.0 mm; 144 sagittal slices were acquired covering the whole brain.

### Data Preprocessing and Network Construction

Data were preprocessed using the DPARSF toolbox (Yan and Zang, 2010) in SPM12^1^. The first 10 functional volumes of each subject were removed from analysis because of the instability of the equipment and the subjects’ adaptation to the environment. Then, images were corrected for section timing and head movement. Infants were excluded with a threshold of 2.0 mm in translation or 2.0° in rotation. Subsequently, the functional images of each subject were coregistered to their corresponding T1WI high-resolution image, and T1WI images were segmented with UNC 2-year-old tissue probability maps (Shi et al., 2011). After that, images were normalized to the 2-year-old brain template (Shi et al., 2011) and resampled to 3 mm × 3 mm × 3 mm. Further steps were performed, including temporal band-pass filtering (0.01–0.08 Hz), linear detrending, and regression of the cerebrospinal fluid signal, white matter signal, whole-brain averaged signal, and head motion parameters in 24 directions.

**FIGURE 1:**
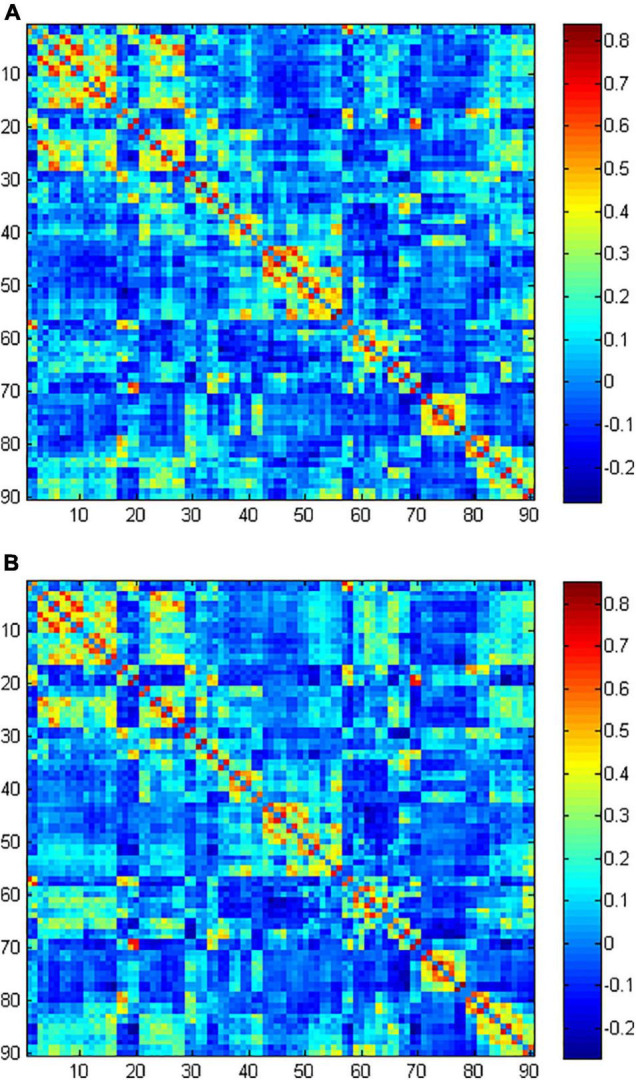
The group-averaged functional connectivity matrix of normal-hearing (NH) group **(A)** and sensorineural hearing loss (SNHL) group **(B)**.

The functional network was constructed and analyzed using GRETNA^2^. The functional images were parcellated into 90 brain regions based on the UNC 2-year-old infant atlas (Shi et al., 2011). Each region was considered as a node of the network. Then, the mean time series of each region were acquired, and Pearson’s correlation coefficients of the mean time series between each pair of nodes were calculated, resulting in a 90 × 90 correlation matrix. Fisher’s R-to-Z transformation was performed for the graph theory analysis. The group-averaged matrix of the two groups were showed in Figure 1.

### Global and Nodal Topological Properties

We used a wide range of sparsity thresholds (0.1 to 0.4, step 0.01) to avoid deviations from using a single-sparsity threshold. The area under the curve (AUC) of both global and nodal network metrics was calculated and compared. Global parameters included small-world parameters (clustering coefficient Cp, characteristic path length Lp, normalized clustering coefficient γ, normalized characteristic path length λ, and small-worldness σ) and network efficiency parameters (local efficiency Eloc and global efficiency Eglob). Cp, γ, and Eloc were used to measure segregation, while Lp, λ, and Eglob were used to measure integration (Rubinov and Sporns, 2010). A network with high Cp and γ, as well as low Lp and λ, was considered to have a small-word organization (Suo et al., 2019). A network was defined as a small-world network when the small-worldness (σ = γ/λ) > 1, which reflects an economic model to transform information efficiently (Achard and Bullmore, 2007). Nodal parameters included nodal degree, nodal efficiency, and nodal betweenness. The details and interpretations of global and nodal parameters mentioned above were explained in a previous study (Rubinov and Sporns, 2010). Hubs are important for information transmission, which is variably defined in the literature. In this study, a node was considered to be a hub in a network if its nodal degree, nodal efficiency, and nodal betweenness were at least 1 standard deviation greater than the mean value (Xu et al., 2016).

### Edge-Wise Connectivity Strength Analysis

The edge-wise connectivity strength difference was evaluated using the network-based statistic (NBS) (Zalesky et al., 2010) by looking for different clusters of connections between the two groups. The initial threshold was set to be a *p*-value less than 0.001. Then, the non-parametric permutation method (5,000 permutations) was used to estimate the statistical significance. Subnetworks with corrected *p*-values less than 0.05 were considered to be significant.

### Statistical Analysis

Demographic data were compared using the Statistical Package for the Social Sciences (SPSS), version 22.0 (IBM Corp., Armonk, NY, United States). Graph theory properties were compared using GRETNA software. Between-group differences in the AUC of global and nodal network metrics were tested using two-sample *t*-test. A *p*-value less than 0.05 was identified as significant. When comparing nodal parameters, the Bonferroni correction was applied to correct for multiple comparisons with a significant *p* < 0.05.

## Results

### Demographic and Clinical Characteristics

Demographic and clinical data for the subjects in the NH group and SNHL group are shown in Table 1. No significant differences were found in age and gender.

**TABLE 1:** Summary of the demographic and clinical data.

	NH group	SNHL group	*p*-value
Number (n)	30	34	–
Age (months)^a^ (mean ± SD)	24.03 ± 7.18	23.41 ± 8.26	0.089
Age range (months)	11-36	11-36	–
Gender (male/female)^b^	16/14	16/18	0.616
ABR of left ear (dB HL)	>90	–	–
ABR of right ear (dB HL)	>90	–	–

*ABR, auditory brainstem response; NH, normal hearing; SNHL, sensorineural hearing loss; SD, standard deviation.*

*^a^Statistical analyses for comparisons between groups were carried out with t-tests.*

*^b^Statistical analyses for comparisons between groups were carried out with χ2 tests.*

### Global and Nodal Topologic Organization of the Functional Network

**FIGURE 2:**
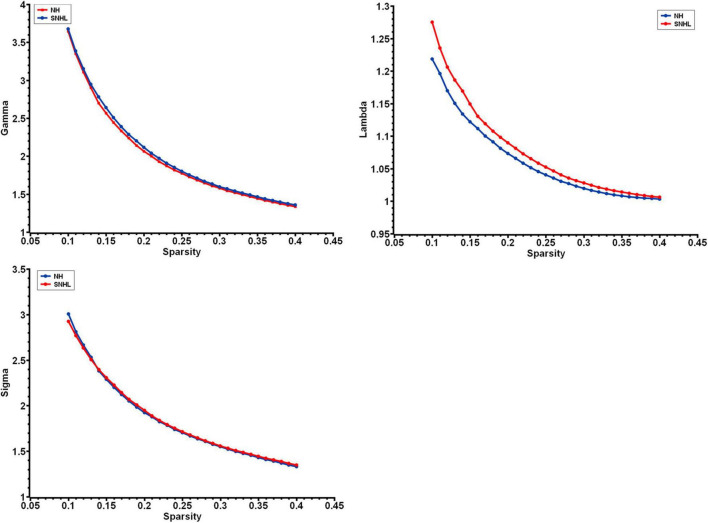
The small-world parameters of functional networks as a function of sparsity threshold from 0.1 to 0.4. Both the NH group and SNHL group exhibited γ, λ, and σ larger than 1, which indicates that both groups exhibited the small-word organization. (NH, normal hearing; SNHL, sensorineural hearing loss; γ, normalized clustering coefficient; λ, normalized characteristic path length; σ, small-worldness).

**FIGURE 3:**
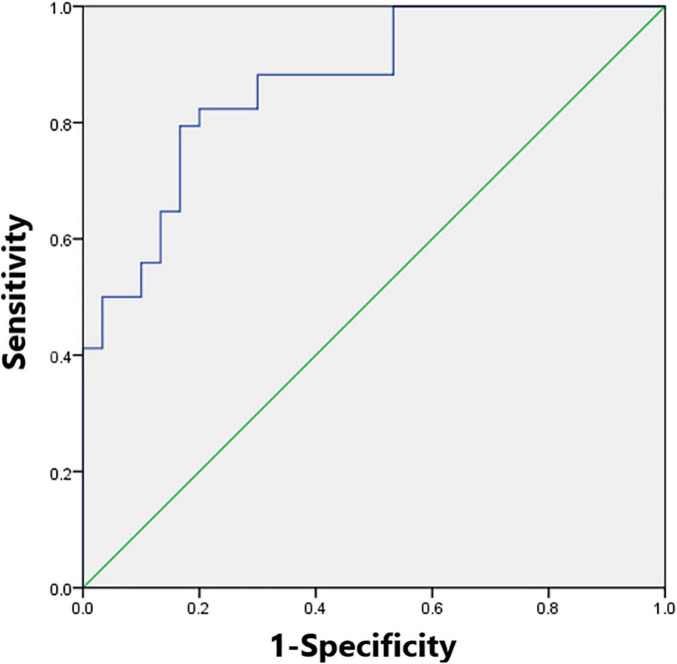
The receiver operating characteristic (ROC) curve based on binary logistic regression using the nodal degree of the left inferior marginal angular gyrus and the bilateral thalamus. The module reached the highest area under the curve of 0.87.

We found λ > 1, γ > 1, and σ > 1 across all the sparsities in both the SNHL group and the NH group (Figure 2). There was no significant difference in the global parameters (Cp, Lp, γ, λ, σ, Eg, and Eloc) of the functional network (Table 2). After Bonferroni correction, compared with the NH group, the SNHL group showed an increased nodal degree in the left inferior marginal angular gyrus and a decreased nodal degree in the bilateral thalamus (Table 3). There was no significant difference in nodal betweenness and nodal efficiency. The receiver operating characteristic (ROC) curve based on binary logistic regression using the nodal degree of the left inferior marginal angular gyrus and the bilateral thalamus was showed in Figure 3.

**TABLE 2:** Global network parameters in NH group and SNHL group.

Global network measures	NH group (*n* = 34)	SNHL group (*n* = 30)	*t*-value	*p*-value
Cp	0.17 ± 0.009	0.17 ± 0.011	−1.332	0.190
Lp	0.53 ± 0.009	0.53 ± 0.018	−1.563	0.125
γ	0.59 ± 0.068	0.60 ± 0.075	−0.466	0.643
λ	0.32 ± 0.004	0.32 ± 0.008	−2.096	0.052
σ	0.55 ± 0.061	0.55 ± 0.072	−0.111	0.912
Eglob	0.17 ± 0.002	0.17 ± 0.004	1.510	0.138
Eloc	0.23 ± 0.005	0.23 ± 0.006	−1.384	0.173

*NH, normal hearing; SNHL, sensorineural hearing loss; Cp, clustering coefficient; Lp, characteristic path length; γ, normalized clustering coefficient; λ, normalized characteristic path length; σ, small-worldness; Eglob, global efficiency; Eloc, local efficiency.*

**TABLE 3:** Regions of altered nodal degree in NH group and SNHL group (*p* < 0.05, Bonferroni corrected).

Brain regions	NH group	SNHL group	*t*-value	*p*-value
THA.L	5.24 ± 1.799	3.37 ± 1.138	4.207	0.00013
THA.R	5.25 ± 1.667	3.55 ± 1.152	3.993	0.00024
IPL.L	6.07 ± 1.390	7.81 ± 1.470	−3.860	0.00037

*NH, normal hearing; SNHL, sensorineural hearing loss; THA.L, left thalamus; THA.R, right thalamus; IPL.L, left inferior marginal angular gyrus.*

### Hubs in the Brain Network

**FIGURE 4:**
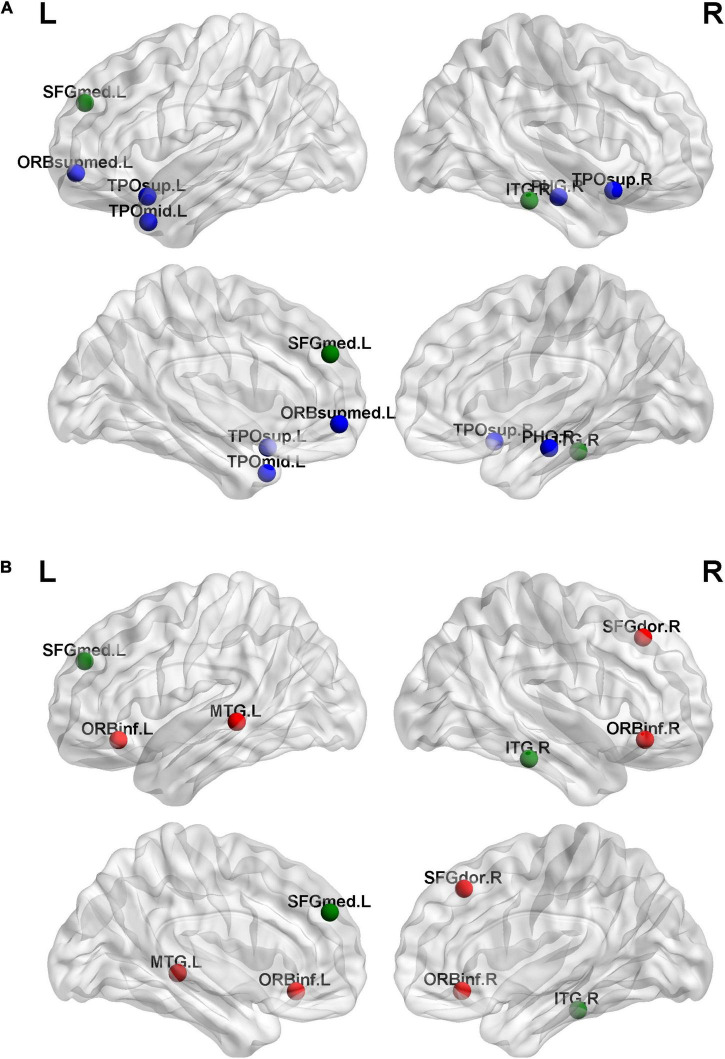
Hubs for brain functional networks in normal-hearing (NH) group **(A)** and sensorineural hearing loss (SNHL) group **(B)**. The hubs identified in both groups are shown in green color. While the hubs for the SNHL group only are present with red color, the hubs for the NH group only are shown in blue color.

We found six hubs in the SNHL group and seven hubs in the NH group. As shown in Figure 4, the left medial superior frontal gyrus and right inferior temporal gyrus were hubs in both the SNHL group and NH group. The right dorsolateral superior frontal gyrus, bilateral orbital part of the inferior frontal gyrus, and left middle temporal gyrus were hubs only in the SNHL group, while the left medial orbital superior frontal gyrus, right parahippocampal gyrus, bilateral temporal pole of the superior temporal gyrus, and left temporal pole of the middle temporal gyrus were hubs only in the NH group.

### Functional Connectivity

**FIGURE 5:**
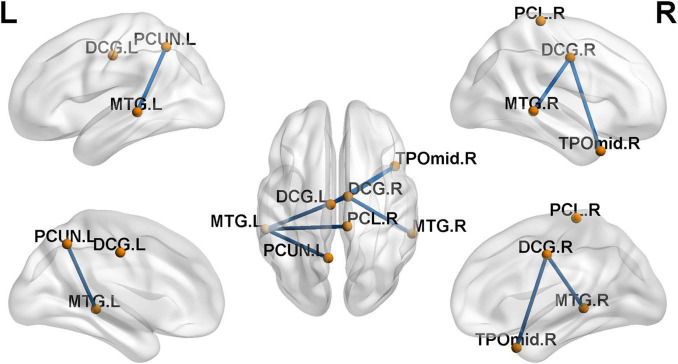
Decreased functional connections in sensorineural hearing loss (SNHL) group when compared with the normal-hearing (NH) group (*p* < 0.001, NBS corrected). The subnetwork is consisted with seven nodes and six edges.

Compared with the NH group, the SNHL group presented a significantly decreased subnetwork component in the NBS analysis results (*p* < 0.001, NBS corrected). As shown in Figure 5, the subnetwork consisted of seven nodes and six edges. The involved regions included the bilateral median cingulate and paracingulate gyri, left precuneus, right paracentral lobule, bilateral middle temporal gyrus, and right temporal pole of the middle temporal gyrus.

## Discussion

This study investigated the complex brain network organization in infants with profound bilateral congenital SNHL within a critical period of development based on rs-fMRI using graph theory analysis. We found differences between the SNHL group and NH group in nodal degree, hub distribution, and whole-brain functional connectivity. These results indicated that functional reorganization occurred in the first few years of hearing deprivation.

### Topological Parameter Alterations

The human brain network exhibits small-world topology from the fetal stage (Turk et al., 2019). In this study, both the SNHL group and NH group showed small-word organization, with no significant differences. This result was consistent with previous studies on sudden unilateral sensorineural hearing loss in adults and prelingually deaf adolescents (Li et al., 2016; Xu et al., 2016), indicating that the efficiency of information transmission in the brain was not affected by hearing deprivation.

However, compared with normal-hearing infants, SNHL infants showed no significant difference in global topological parameters, which was consistent with the study of Zhang et al. (2018) on long-term unilateral hearing loss but different from the study of Xu et al. (2016) on sudden unilateral sensorineural hearing loss. Alteration of functional network global topological parameters has been observed in many neurological disorders, including epilepsy (Zhang et al., 2011; Li et al., 2020), Alzheimer’s disease (Pereira et al., 2016), Parkinson’s disease (Suo et al., 2017b), and essential tremor (Benito-León et al., 2019). These patients usually have obvious neurologic symptoms, including cognitive impairment or motor disorder, with a long duration. The duration of sudden sensorineural hearing loss is less than 3 days, which was significantly shorter than that of patients in Xu et al.’s study (at least 2 months) and infants with congenital hearing loss in our study. Furthermore, SNHL patients usually do not have neurological symptoms as severe as those with neurological disorders. Based on the above analysis, we speculate that the sudden loss of sensory import could change the pattern of information transmission of the functional network, but the brain will adjust to adapt and compensate for the loss of sensory in order to maintain the topological organization if the situation persists over a long period of time. This may be a result of neuroplasticity. Moreover, considering the special age of our participants, the maintenance of global topological organization may be the basement of the maximal plasticity in the critical period, which leads to the rapid reconstruction of hearing and speaking ability after CI.

In the bilateral thalamus, a decreased nodal degree was observed in the functional network of SNHL infants, which means less or decreased functional connectivity with other regions. This finding likely underlies the key role of the thalamus in auditory information transfer. The thalamus, which consists of many nuclei, is the relaying nucleus of the sensory pathway and receives input from the cortex (Hwang et al., 2017; Martini et al., 2021). It also mediates important functions including memory, emotion, and attention (Arend et al., 2015; Geier et al., 2020). Imaging evidence has also shown decreased thalamic functional connectivity with both auditory and non-auditory regions in long-term SNHL patients (Xu et al., 2019). Our finding was consistent with Xu et al.’s (2019) result. Moreover, we found an increased nodal degree in the left inferior marginal angular gyrus, which was included in the inferior parietal lobule. The inferior parietal lobule was associated with sensory integration, body image, self-concept, and executive function (Torrey, 2007). The ICA method has also found the inferior parietal lobule to be included in the DMN, the frontoparietal control network, and the cingulo-opercular network (Igelström and Graziano, 2017). In the study of Luan et al. (2019), the right inferior parietal lobule showed increased intra-network connectivity within the right frontoparietal network, and this alteration was correlated with the Symbol Digit Modalities Test scores of SNHL patients. The increased nodal degree in the inferior marginal angular gyrus might be related to the alteration of high-order congenital functions in SNHL infants.

### Hub Distribution Differences

In a brain network, regions that are more interconnected with other regions and possess a high nodal degree are considered as hubs (Xu et al., 2016; Medaglia, 2017; Zhao et al., 2019). Hub changes are considered to be related to neurological and psychological diseases (Tian et al., 2016; Lin et al., 2018). We found fewer hubs in infants than a previous study in adults using the same method to identify hubs (Xu et al., 2016), which may be due to the fact that the number of hubs increases with age (Cao et al., 2017). In this study, the hubs of both the SNHL group and NH group were located in the prefrontal lobe and temporal lobe. This distribution was consistent with previous studies on the early developing brain (Grayson and Fair, 2017; Oldham and Fornito, 2019; Turk et al., 2019; Wen et al., 2019).

The middle temporal gyrus is involved in both auditory and visual processes, namely word processing and action observation (Van Essen et al., 1986; Papeo et al., 2019). The middle temporal gyrus acting as a hub in the SNHL group in our study might be a result of deaf infants relying more on visual information. However, the functional connectivity between the thalamus and middle temporal gyrus was previously found to be decreased in SNHL adults (Xu et al., 2019). The cortical thickness of the middle temporal gyrus was found to be increased in postlingually deaf adults (Pereira-Jorge et al., 2018) but decreased in prelingually profound SNHL children (Qu et al., 2020) and postlingually deaf adults (Sun et al., 2021). Thus, we speculated that the functional and structural changes in the middle temporal gyrus in SNHL patients will differ between children and adults, and between prelingually deaf patients and postlingually deaf patients. The mechanism of the alterations in the middle temporal gyrus in different conditions needs to be studied further. The parahippocampal gyrus is a key region of memory formation (Düzel et al., 2003). The parahippocampal gyrus and temporal pole are involved in auditory memory (Muñoz-López et al., 2015; Córcoles-Parada et al., 2019). In the study of Rönnberg et al. (2011), hearing loss patients showed impaired episodic memory. Accordingly, in another study, the gray matter volume of the right parahippocampal gyrus was found to be decreased in unilateral hearing loss patients (Yang et al., 2014). In our study, the parahippocampal gyrus and temporal pole were hubs only in the NH group. The absence of these two regions as hubs in the SNHL group suggested weakened auditory memory ability, which could be a consequence of hearing deprivation.

### Functional Connectivity Differences

The main regions involved in the decreased subnetwork of the SNHL group were the DMN, auditory network, and sensorimotor network. Decreased functional connectivity between the auditory and motor regions in hearing loss patients has been reported by previous studies, which was considered a result of failure in imitating speech sounds due to hearing deprivation (Shi et al., 2016; Bonna et al., 2021). In our study, we detected a weaker connection between the middle temporal gyrus and paracentral lobule. The middle temporal gyrus is a key region of auditory perception and is involved in word processing (Papeo et al., 2019; Feng et al., 2021). Clinical studies also found poor balance and gait performance of SNHL children (Melo, 2017; Melo et al., 2018; Sokolov et al., 2019). Decreased functional connectivity between the middle temporal gyrus and paracentral lobe may reflect the separation of auditory and motor function in SNHL infants.

We also found decreased functional connectivity between the DMN and auditory regions. Similar changes were also found in a previous study of Li et al. on congenital severe sensorineural hearing loss infants under 2 years old (Li et al., 2019). The DMN is identified in infancy and becomes similar to that in adults at 2 years of age (Gao et al., 2009, 2015). The functional connectivity between DMN regions was found to increase with age during childhood and adolescence (Fan et al., 2021). The DMN consists of brain regions that are more active when there are no external tasks (Anticevic et al., 2012). It is involved in multiple high-order cognitive functions, including self-related cognition, working memory, and emotion processing (Buckner et al., 2008; Anticevic et al., 2012; Chai et al., 2014). Moreover, the development of theory of mind, which is considered to be correlated with the functional connectivity strength of DMN regions, was found to be affected by the loss of linguistic experience caused by hearing loss (Hughes et al., 2019; Richardson et al., 2020). Decreased functional connectivity within the DMN was found in a previous ICA study by our team (Wang et al., 2021). Combined with the findings of Wang et al. (2021), this suggests that decreased functional connectivity between DMN regions and auditory regions and within DMN regions may suggest impaired high-order cognitive functions caused by hearing deprivation in the developing brain of SNHL infants.

### Limitations

There are several limitations to the present study. First, the small sample size may decrease the statistical power of the results. Second, the human brain develops rapidly in the first few years of life, and there may be individual differences in brain development due to the impact of living environment. Third, there is no universal infant atlas, and using different templates can affect the accuracy of the results. These factors may reduce the detection power. Furthermore, infants who participated in the present study were sedated with chloral hydrate, which is usually thought to affect the resting-state functional connectivity compared with natural sleep. However, previous studies in SNHL infants using rs-fMRI under sedation have delivered meaningful results, which showed the feasibility of the use of sedation (Xia et al., 2017; Li et al., 2019; Wang et al., 2019). Moreover, Fransson et al. (2007) showed that there was no significant effect on functional connectivity in infants and children under sedation during scanning. Another factor which must be considered is that although we have used the hearing protection measure, the NH group was still exposed to a greater level of scanner noise than the SNHL group due to massive threshold shifts in the SNHL sample. This may bring some inevitable confounding factors. Finally, the results drawn from graph theory analysis are often not intuitive and may be difficult to interpret (Lv et al., 2018). In future studies, the sample size should be increased and further grouped according to age. Combined studies using other methods are needed as a complementary. Long-term follow-up should be conducted to explore the value of complex network analysis as a biomarker for predicting CI outcomes.

## Conclusion

This study used graph theory analysis based on fMRI to investigate alterations of brain functional networks in profound bilateral congenital SNHL in infants in a critical period of development. We found that the functional brain network of SNHL infants within the critical period still maintains the balance of integration and segregation. Compared with NH infants, we found an increased nodal degree in the left inferior marginal angular gyrus and decreased nodal degree in the bilateral thalamus in SNHL infants. We also found a different hub distribution and functional connectivity in both auditory regions and high-order cognitive regions in SNHL infants. These changes reflect a functional network reorganization and potential changes in high-order cognitive function in SNHL infants. This study also provided novel insights into functional network alterations in the early stage of profound bilateral congenital SNHL.

## Data Availability Statement

The datasets presented in this article are not readily available because the data also forms part of an ongoing study. Requests to access the datasets should be directed to GF, fanguog@sina.com.

## Ethics Statement

The studies involving human participants were reviewed and approved by Medical Science Research Ethics Committee, The First Affiliated Hospital of China Medical University. Written informed consent to participate in this study was provided by the participants’ legal guardian/next of kin.

## Author Contributions

WC, SW, and GF designed the study. WC and SW performed the research. WC and BC analyzed the data. WC wrote the draft. All authors contributed to the article and approved the submitted version.

## Conflict of Interest

The authors declare that the research was conducted in the absence of any commercial or financial relationships that could be construed as a potential conflict of interest.

## Publisher’s Note

All claims expressed in this article are solely those of the authors and do not necessarily represent those of their affiliated organizations, or those of the publisher, the editors and the reviewers. Any product that may be evaluated in this article, or claim that may be made by its manufacturer, is not guaranteed or endorsed by the publisher.

## Footnotes

1
http://www.fil.ion.ucl.ac.uk/spm/software/spm12/

2
http://www.nitrc.org/projects/gretna/
